# Supplementary material for: Ectopic Overexpression of Maize Heat Stress Transcription Factor ZmHsf05 Confers Drought Tolerance in Transgenic Rice
Source: Genes (Basel). 2021 Oct 1;12(10):1568. doi: 10.3390/genes12101568 (PMC8536174; doi:10.3390/genes12101568)
Supplement: Supplementary file 1 [file genes-12-01568-s001.zip › genes-1387533-supplementary.pdf]

Supplementary material for

Heat Shock Transcription Factor05 of *Zea mays* L., a Key Drought-related Gene Involving Abscisic acid-dependent Pathway

Table S1. The sequence of primers used in this study

Use	Name	Forward Primer (5'-3')	Reverse Primer (5'-3')
1301a	*ZmHsf05*	GGGGTACCATGAGC CACGGGAACGGGAT	GCTCTAGACTAATGTCT GGGTCCATGTTGTTTC
1305-GFP	*ZmHsf05*	GCTCTAGAATGAGC CACGGGAACGGGAT	TCCCCCGGGATGTCTG GGTCCATGTTGTTT
RT-qPCR	*ZmHsf05*	GAGTGCCGCCAACA ATA	CCACCTTTCTGAAGCC ATA
	ZmActin1	GGGATTGCCGATCG TATGAG	GAGCCACCGATCCAGA CACT
pGBKT7	*ZmHsf05*	ATGGCCATGGAGGC CGAATTCATGAGCC ACGGGAACGGG	CCGCTGCAGGTCGACG GATCCCTAATGTCTGGG TCCATGTTGTTT
Semi-quantitative	*ZmHsf05*	TCGCCGCCGTTCCTT ACCAA	CATATCCAAAGTGTCCT ACC
	OsActin	CTGACGGAGCGTGG TTACTCAT	TGGTCTTGGCAGTCTC CATTTC
ABA-related and stress-related genes	*OsLEA3*	ATACCAAGGAGGCG ACGAAGG	TCATCCCCAGCGTGCT CATCA
	OsASR5	ACCACCTGTTCCAC CACAAGA	TCTTCTCGTGGTGCTCG TGGA
	OsNCED3	CGCAACAGTAAAAA GAATTAACAGC	TATACACACACGCGGT CGTT
	OsAP3	ACGTCCAGCAACGC ATCCTTAC	TCATACGGTATCCAGC CTCAGC

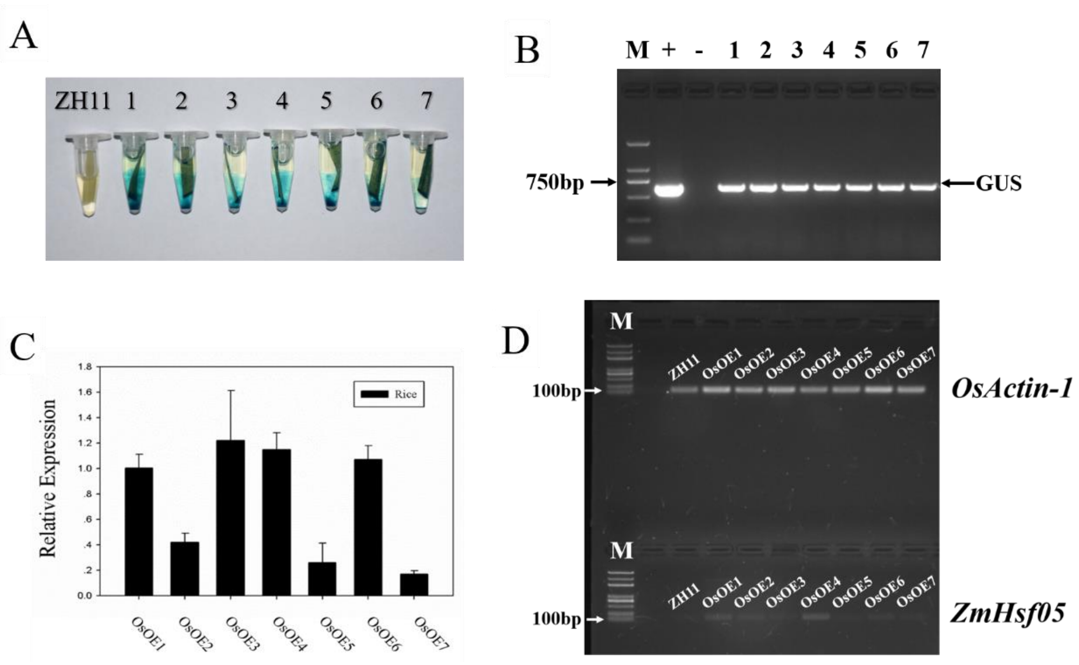

Supplemental Figure S1. Identification of positive transgenic rice seedlings overexpressing *ZmHsf05* gene. (A) GUS staining of transgenic rice leaves. (B) GUS gene PCR of transgenic rice. (C) Quantitative detection of *ZmHsf05* gene expression in transgenic rice. (D) Semi-quantitative detection of *ZmHsf05* gene expression in transgenic rice. The *OsActin-1* gene was used as the internal control for normalization. M stands for maker. Vertical bars indicate means ± S.E. ($n \geq 3$).
